# Decentralization and Hospital Pharmacy Services: The Case of Iranian University Affilliated Hospitals

**Published:** 2013

**Authors:** Reza Ashna Delkhosh, Ali Ardama, Jamshid Salamzadeh

**Affiliations:** a *Clinical Pharmacy Department, School of Pharmacy, Shahid Beheshti University of Medical Sciences, Tehran, Iran.*; b *Pharmaceutical Sciences Research Center, Shahid Beheshti University of Medical Sciences, Tehran, Iran.*; c *Students Research Committee, School of Pharmacy, Shahid Beheshti University of Medical Sciences, Tehran, Iran.*

**Keywords:** Pharmacy services, Hospital, Privatization, Satisfaction

## Abstract

The aim of this study was to evaluate the satisfaction rate of hospital managerial/clinical teams (HMCTs) including principles (chief executives), managers, supervisor pharmacists and head nurses from services presented by private sectors directing 10 pharmacy departments in hospitals affiliated to Shahid Beheshti University of Medical Sciences.

This study is an observational and descriptive study in which a questionnaire containing 16 questions evaluating the satisfaction of the HMCTs from private sectors, and questions about demography of the responders was used for data collection. Collected data was applied to assign a satisfaction score (maximum 64) for each respondent. SPSS 17.0 and Microsoft Office Excel 2007 were used for statistical description and analysis of these information (where applicable). Overall, 97 people in charge of the hospitals (HMCTs) entered the study. The average satisfaction score was 26.38 ± 6.81 with the lowest satisfaction rate observed in Mofid children specialty hospital (19.5%) and the highest rate obtained for Imam Hussein (p.b.u.h) general hospital (65.3%). Generally, 59% of the HMCTs believed that the function of the private sector in the pharmacy of hospitals is satisfactory.

Assuming that the satisfaction scores under 75% of the total obtainable score (*i.e. *48 out of 64) could not be considered as an indicator of desired pharmacy services, our results revealed that the status of the services offered by private sectors are far behind the desired satisfactory level.

## Introduction

Decentralization is primarily related to devolution of authority (1) and has been promoted by the supporters of health sector reform in developing countries for decades. It is commonly accepted that decentralization in government-provided health services to sub-national levels is necessary to produce an optimum combination of top-down and bottom-up planning (2). Four major analytical frameworks that have been used by authors who address problems of decentralization in the health sector are as follows: public administration, local fiscal choice, social capital and principal agent approaches (2). Public administration could be presented in special forms including deconcentration, delegation, devolution and privatization. Deconcentration is defined as shifting power from the central offices to peripheral offices of the same administrative structure. Delegation shifts responsibility and authority to semi-independent agencies and devolution shifts responsibility and authority from the central offices of the Ministry of Health to separate administrative structures still within the public administration. Privatization relocates operational responsibilities and, in some cases, ownership from government to private sectors, usually with an official agreement to describe what is expected in exchange for public funding (4). A central issue of the public administration approach has been designed to define the appropriate levels for decentralizing functions, responsibility and authority (5).

Decentralization can have considerably different impact on the process of program development and delivery. A report by the United Nations Population Fund, published in June 2000, reveals that “decentralization has emerged as a result of a global trend to local autonomy and self determination and as a result of a trend to reduce reliance on centralized planning of economies and be more responsive to market forces as well as local needs and characteristics”. This report also introduces the decentralization as an evolving political and administrative process rather than a particular form of organizational structure or institutional arrangement. This means that the characteristics of decentralization in any particular country are dynamic and are subjected to rapid change depending on the agenda of current government in power and popular trends (6). 

There have been several attempts at reforming the health system in Iran, but an organized effort has not been made since 2002. A number of initiatives and studies to develop tools for improving health system performance were consolidated and a health sector reform project was designed that has been funded by the World Bank. Drawing from the principle in Article 29 (Welfare benefits) of the constitution of the Islamic Republic of Iran that emphasizes all citizens have the right to enjoy health services and medical care, the objectives are as follows: I: Designing and testing a universal basic minimum health services package and strengthening patient referral system, ensuring a better quality health services that are responsive to the needs of the communities. II: Assuring the stewardship and good governance in the public sector health system guaranteeing the pro-poor policies. III: Improving health planning and management including decentralization in the health sector by delegating the administrative and financial authority. IV: Reviewing the existing health financing options for introducing measures to assure fair financing, eliminating inefficiencies and bringing equity. V: Making organizational arrangements for conceptualizing, formulating and implementing health sector reforms (7) From 2006, in compliance with the objectives of the fourth as well as the fifth Iranian National Development Plan, in order to improve the quality of the services offered by health system, and also in accordance with the decentralization initiatives, in particular privatization in the health sector, private parties were entered to the pharmaceutical industry (8, 9) as well as to the hospitals to direct the pharmacy departments. Shahid Beheshti University of Medical Sciences was one of the pioneer universities that have started privatization plans in its affiliated hospitals. Pharmacy departments were among the first units considered to outsource the operational responsibilities of the pharmacies. The current study is an approach to clarify the benefits or drawbacks of this process using the satisfaction rate of managerial/clinical teams in the relevant hospitals.

## Experimental

*Methods*

This observational and descriptive study was conducted in 10 hospitals of Shahid Beheshti University of Medical Sciences from November 2009 to July 2010, in which 97 hospital principles, managers, supervisor pharmacists and head nurses were included. First of all, a questionnaire containing the following two parts was constructed: I: Demographic information of participants. II: Sixteen questions to evaluate the satisfaction rate of hospital managerial/clinical teams (HMCT).

In the first phase, the primary questionnaire underwent a face validity evaluation by 7 pharmacy and pharmacoeconomic expert managers as well as faculty members, and was finalized for applying in this study (Table 1).

**Table 1:** Study questionnaire

**No**	**Question**	**agree**	**Completely** **agree**	**disagree**	**Completely** **disagree**
1	The drug (medicine) shortage related problems have been decreased after privatization of the hospital pharmacy.				
2	Medical equipment’s and medical supplies related problems have been decreased after privatization of the hospital pharmacy.				
3	The number of prescriptions supplied from the out of the hospital has been decreased after privatization of the hospital pharmacy.				
4	The numbers of prescriptions for getting the medical equipments and medical supplies out of the hospital have been decreased after privatization of the hospital pharmacy.				
5	The quality of services provided by the pharmacist has been increased after privatization of the hospital pharmacy.				
6	Regularity and discipline on distribution of drugs and medical equipment to the wards have been improved after privatization of the hospital pharmacy.				
7	Pharmacy errors due to poor performance have been decreased after privatization of the hospital pharmacy.				
8	Problems due to inappropriate medicine supply have been reduced in the afternoonand night hours after privatization of the hospital pharmacy.				
9	The quantity of drugs stocked in the wards declined after privatization of the hospital pharmacy				
10	The prescription pricing errors and extortion have been declined after privatization of the hospital pharmacy				
11	Non-pharmacy related problems of hospital have been declined after privatization of the hospital pharmacy.				
12	The insurance prescriptions deduction has been decreased after privatization of the hospital pharmacy				
13	The activities of the Drug and Therapeutic Committee have been increased after privatization of the hospital pharmacy.				
14	The role of pharmacy as a drug information center have been improved after privatization of the hospital pharmacy				
15	The role of hospital logistics department to supply drugs and equipments has been declined after privatization of the hospital pharmacy.				
16	Overall, the performance of pharmacy department has improved after privatization of the hospital pharmacy.				

In the second phase of the study, the questionnaires were given to each HMCT member and their responses were collected for analysis. A satisfaction score (max. 64) was assigned

for each respondent based on their answer to the questions. Collected data was entered the Microsoft office Excel 2007 and the Statistical Package for Social Sciences (SPSS^®^, version 17.0) programs. Appropriate statistical tests (significance level of < 0.05) were used whenever applicable. In order to evaluate the relationship between the satisfaction of HMCT and features of each hospital, Pearson correlation was applied. In addition, to compare the differences between satisfaction score of the hospital managerial team (principles and managers) and hospital clinical team (supervisor pharmacist and head nurses) the student t-test was applied. 

## Results

Overall 97 people (77 women and 20 men) in charge of 10 hospitals (Ayatollah Taleghani, Mofid Children, Shohada-e Tajrish, Mahdieh, Imam Hussein (p.b.u.h), Ayatollah Modarres, Loghman Hakim, 15^th^ Khordad, 3^rd^ Sh’aban-e Damavand and Mofateh-e Varamin) entered the study. 

Among these 97 HMCTs, 7 were chief executives, 10 supervisor pharmacists, 10 managers and 70 nurses. The age of the participants was between 25 and 60 years and in terms of education, the principles were specialist in various fields of medicine, the managers had a bachelor’s degree or higher, the supervisor pharmacists were Pharm. D. and the nurses had a bachelor’s degree.

Table 2 presents the features of each hospital in terms of number of active beds, number of wards and total bed-day occupancy rate per one year. In addition, Table 3 represents the scores that each group of HMCT achieved, separately.

**Table 2:** Characteristics of study hospitals

**Hospitals**	**Active Ward**	**Active Bed**	**Total bed-day occupancy in one yea**r
Imam Hussein	19	461	138686
Ayatollah Modarres	12	264	65188
Mofid	14	222	66467
Shohada-e Tajrish	16	339	90671
Loghman Hakim	18	355	100488
Mahdieh	6	149	31226
15^th^ Khordad	6	97	22986
3^rd^ Sha’ban	6	46	7642
Ayatollah Taleghani	20	422	109866
Ayatollah Mofateh	7	108	24632

**Table 3:** Mean ± SD satisfaction scores assigned by HMCTs to private sectors

**HMCT**	**Mean ± SD Scores**
Hospital Managers	33.33 **± **5.37
Principles (chief exec.)	32.25 ± 6.45
Supervisor Pharmacists	21.37 ± 8.21
Nurses	21.31 **± **7.61

SD: Standard Deviation; HMCT: Hospital Managerial/Clinical Team

*The relationship between total satisfaction scores of the hospital managerial team and clinical team*

**Figure 1:**
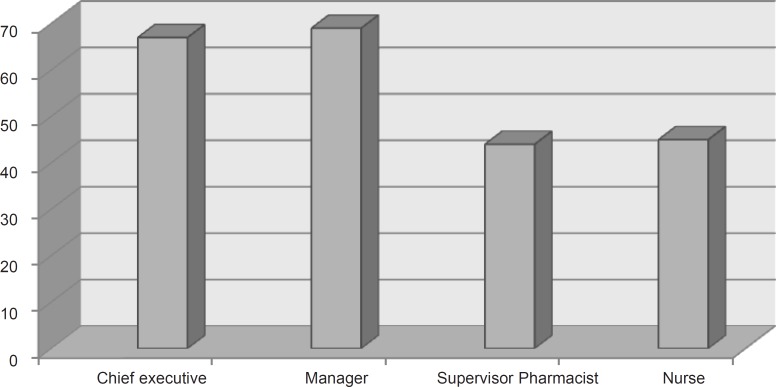
Average satisfaction rate (%) of HMCTs from private sector in pharmacy departments.

The total *satisfaction *score of the managerial team (hospital principles and managers) was 32.90 ± 5.80 (out of maximum score of 64), whereas the total *satisfaction *score of clinical team (supervisor pharmacist and head nurses) was 21.86 ± 8.71, which shows a significant statistical difference between these two groups (p < 0.0001, 95% CI: 5.42 ± 16.66). As shown in Figure 1, the clinical team had a lower average rate of satisfaction about the services provided by private sector than that of the managerial team.

*The satisfaction scores that were achieved by each hospital*

As shown in Table 4, the average satisfaction score was 26.38 ± 6.81 with the lowest satisfaction rate observed in Mofid children›s specialized hospital (19.5%) and the highest rate obtained for Imam Hussein (p.b.u.h) general hospital (65.3%).

**Table 4:** The average satisfaction scores for each hospital

**Hospital**	**Achieved Point**	**Obtainable Point**	**Percentage %**
Imam Hussein	251	384	65.3
Ayatollah Modarres	166	288	57.6
Mofid	103	528	19.5
Shohada-e Tajrish	428	912	46.9
Loghman Hakim	188	336	55.9
Mahdie	334	672	49.7
15^th^ Khordad	143	336	42.5
3^rd^ Sha'ban	180	336	53.5
Ayatollah Taleghani	306	672	45.5
Ayatollah Mofatteh	132	240	55

*The relationship between the total satisfaction score and the characteristics of the hospitals*

The results showed that there was no meaningful and significant relationship between the total satisfaction scores and the features of hospitals including: number of active beds (p = 0.48, r = 0.26), number of active wards (p = 0.78, r = 0.10) and the total rate of occupied bed-days in one year (p = 0.53, r = 0.23).

## Discussion

Our study showed that, although most of the HMCTs agreed on improvement of services provided by private sector (according to the responses to each question individually), the average percentage of the satisfaction is not considerably higher than the ones who did not agree with this improvement. Particularly, in some points of views like “increasing faults in dispensing prescriptions”, “decreasing problems of hospital in other areas except pharmacies”, “increase in the activity of pharmaceutical committee”, and “responsibility of pharmacist in responding to drug-related questions of nurses and patients”, the scores were not in favor of the privatization. Moreover, as mentioned before, the hospital clinical team (supervisor pharmacists and nurses), who were in close touch with the pharmacy-related problems and could strictly monitor the medication-related limitations, had a lower satisfaction from the pharmacy services provided by private sectors compared to the hospital administrative teams (hospital principles and managers), who were mainly involved in financial and managerial problems of the hospitals. This might be due to the fact that the monetary profit provided by private sector, as monthly rent and/or a predefined percent of pharmacy sale cited in the contract, is in the first priority for administrative teams of the hospitals helping them to overcome budgetary shortage, not granted by governmental resources.

**Figure 2:**
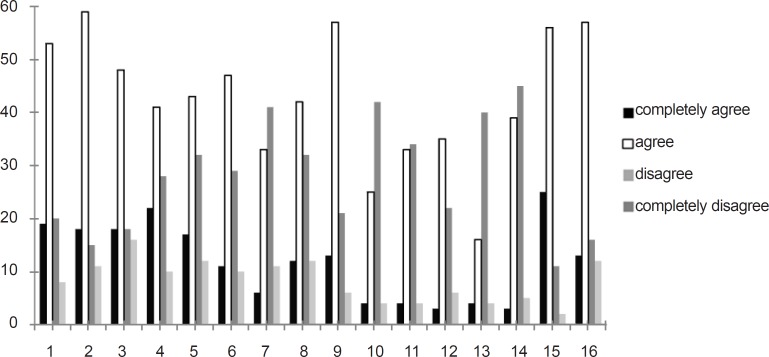
Answers of interviewees to each individual question

 As shown in Figure 2, 50-60% of the respondents agreed on the improvement after privatization and 40-50% thought otherwise. Assuming that satisfaction scores under 75% of the total obtainable score (*i.e. *48 out of 64) could not be considered as an indicator of desired pharmacy services, our results revealed that the status of the services offered by private sectors are far behind the satisfactory level in all types of the studied hospitals (specialized or general; high or low workload).

 In other words, the main goal of privatization, which should be inducing a significant increase in the quality and quantity of pharmacy-related services, was not attained adequately according to the findings of this study. 

In conclusion, the process of privatization in pharmacies, irrespective of the type (specialized or general) and workload of the hospitals (in terms of occupied bed-days), should be reassessed. Moreover, it is necessary to have a regular evaluation of the private sector activities in hospitals to improve the quality and quantity of their services**.** In future researches, evaluating the violation of current good hospital pharmacy practice law by private sectors is also recommended.
